# Effects of silencing the ATP-binding cassette protein E1 gene by electroporation on the proliferation and migration of EC109 human esophageal cancer cells

**DOI:** 10.3892/mmr.2015.3512

**Published:** 2015-03-19

**Authors:** XIAO-RUI LI, LIU-ZHONG YANG, XIAO-QING HUO, YING WANG, QING-HUI YANG, QING-QIN ZHANG

**Affiliations:** Department of Oncology, The First Affiliated Hospital of Xinxiang Medical College, Weihui, Henan 453100, P.R. China

**Keywords:** esophageal cancer, ATP-binding cassette protein E1, electroporation, EC109, cell proliferation

## Abstract

In the present study, the gene expression of ATP-binding cassette protein E1 (ABCE1) in the EC109 human esophageal cancer cell line was silenced using electroporation to examine the effect if the ABCE1 gene on the growth migration and cell cycle of cancer cells. The small interference (si)RNA sequence of ABCE1 was designed and synthesized to transfect the EC109 cells by electroporation. The mRNA and protein expression levels of ABCE1 were then detected by reverse transcription quantitative polymerase chain reaction (RT-qPCR) and western blot analysis. The analysis of the cell cycle and apoptosis was performed using flow cytometry. The effect of silencing the ABCE1 gene on the proliferation, migration and invasive ability of the EC109 human esophageal cancer cells were assessed using a Cell counting kit-8 (CCK-8) and with proliferation, wound-healing and cell invasion assays. The mRNA and protein expression levels of ABCE1 were significantly lower in the experimental group compared with the control group (P<0.05). The apop-totic rate of the experimental group was markedly higher than the control group and blank group (P<0.01). The CCK-8 proliferation assay revealed that, compared with the control and blank groups, the proliferation of the EC109 cells in the experimental group was significantly inhibited (P<0.05). The wound healing assay revealed that the migration capacity of the cells in the experimental group was significantly decreased (P<0.05). The Transwell chamber assay demonstrated that the invasive ability of the EC109 cells in the experimental group was significantly decreased (P<0.01). These results revealed that ABCE1 is closely associated with cell proliferation, invasion and migration in esophageal cancer and silencing the ABCE1 gene by electroporation can significantly reduce the proliferation, invasion and migration capacity of EC109 cells *in vitro*.

## Introduction

Esophageal cancer is one of most common types of gastrointestinal malignant tumor in humans. The incidence of the disease is higher in developing countries and the majority of tumors are squamous carcinoma (1). Although chemotherapy and adjuvant chemotherapy are widely used the treatment of esophageal cancer, the prognosis remains poor, particularly in patients with clinical migration and tumor recurrence (2,3). Previous studies have demonstrated that ATP-binding cassette protein E1 (ABCE1) is important in tumor development and abnormal expression may be associated with malignant tumor proliferation and migration (4,5). In the present study, silencing the ABCE1 gene of EC109 human esophageal cancer cells by electroporation was performed to investigate the effect of the ABCE1 gene on the biological behavior of oesophageal cancer cells and to provide an experimental basis for ABCE1-targeted gene therapy in esophageal cancer.

## Materials and methods

### Cells and reagents

The EC109 human esophageal cancer cells were purchased from the China Center for Type Culture Collection (Wuhan University Collection Center, Wuhan, China). Dulbecco’s modified Eagles’s medium (DMEM), Opti-MEM, TRIzol and trypsin were purchased from Invitrogen Life Technologies (Carslbad, CA, USA). Fetal bovine serum (FBS) was from Gibco-BRL (Carlsbad, CA, USA); plasmid pcDNA-3.1 was from Invitrogen Life Sciences and the DNA marker and reagents for reverse transcription quantitative polymerase chain reaction (RT-qPCR) were purchased from Takara Bio, Inc. (Shiga, Japan). The cDNA synthesis kit was from Toyobo, Co., Ltd. (Osaka, Japan); the AMV reverse transcription kit was from Hangzhou Bioer Techonology Co, Ltd (Hangzhou, China); the small interference (si)RNA was synthesized by Zhuhai Yingping Biotechnology Co, Ltd. (Zhuhai, China) and the PCR primer sequences were synthesized by Shanghai Genepharma Co, Ltd. (Shanghai, China). The cell counting kit-8 (CCK-8) was purchased from Beyotime Intitute of Biotechnology (Shanghai, China); the rabbit anti-human ABCE1 polyclonal and mouse anti-GAPDH monoclonal antibodies were purchased from Santa Cruz Biotechnology, Inc. (1:1,000; Dallas, TX, USA); horseradish-peroxidase (HRP)-labeled rabbit anti-goat immunoglobulin G (1:5,000) was purchased from Beijing Zhongshan Golden Bridge Biotechnology Co, Ltd. (Beijing, China). The enhanced chemiluminescence (ECL) kit was from Thermo Fisher Scientific (Waltham, MA, USA); Matrigel gel was from BD Biosciences (Franklin Lakes, NJ, USA) and the polyvinylidene difluoride (PVDF) membrane was purchased from Bio-Rad Laboratories, Inc. (Hercules, CA, USA).

### siRNA design and synthesis

Based on the ABCE1 cDNA sequence in GenBank (https://www.ncbi.nlm.nih.gov/genbank/), the Basic Local Alignment Search Tool (http://blast.ncbi.nlm.nih.gov/Blast.cgi) was used to design two pairs of oligonucleotide sequences as described in the previous study (6), which were then synthesized by Zhuhai Yingping Biotechnology Co, Ltd. and were as follows: Forward 5′-ATCCGCTACAGCGAGTACGTTTACCTGTGAAGCCACAGATGGGGTAAACGTACTCGCTTAGCTTTTTTG-3′ and reverse 5′-AATFCAAAAAAGCTACAGCGAGTACGTTTACCCCATCTGTGGCTTCACAGGTAAACGTACTCGCTGTAGCG-3′. The negative control siRNA was designed as forward 5′-GATCCGCGAGACCTCAGTATGTTACCTGTGAAGCCACAGATGGGGTAACATACTGAG GTCTCGCTTTTTTG-3′; and reverse 5′-AATTCAAAAAAGCGAGACCTCAGTATGTTACCCATCTGTGGCTTCACAGGTAACATACTGAGGTCTCGCG-3′. The EC109 esophageal cancer cells were cultured in DMEM containing 10% FBS and incubated in a closed incubator in 5% CO_2_ at 37°C for propagation.

### Transfection of cells with the ABCE1-siRNA expression vector

Cells in the logarithmic phase were selected to prepare a single cell suspension. The cells were washed with PBS, digested with trypsin and centrifuged at 12,000 × g for 5 min. Electroporation was performed using the Bio-Rad Gene Pulser Xcell electroporation system (Bio-Rad Laboratories, Inc, Hercules, CA, USA). The cells were added to the electroporation liquid for resuspending, centrifuged at 12,000 × g for 5 min and washed three times in electroporation liquid, retaining the centrifugal sediment. The deposit was resuspended in electroporation liquid, which was placed in an electric cup with 10 *μ*g of the plasmid. Following mixing and placing in an ice-bath for 30 min, electroporation was performed at a voltage of 450 V/cm and electrical capacity of 25 *μ*F for 0.9 ms. The cells were left at room temperature for 30 mins and were then cultured in petri dishes with DMEM containing 10% FBS and 1% double antibiotic (100 IU/ml penicillin, 100 *μ*g/ml streptomycin; Sigma-Aldrich, St. Louis, MO, USA) in a closed incubator in 5% CO_2_ at 37°C. Electrotransfection of cells using an empty vector was performed as a control and untreated EC109 cells were used as a blank control. The effect of RNA interference was determined 48 h after transfection.

### RT-qPCR detection

The cells (1×10^7^) in each group were collected and washed three times in PBS. The total RNA of each group was extracted using TRIzol reagent and reverse-transcribed to cDNA for qPCR amplification. GAPDH was used as a control gene. The ABCE1 primer sequences were as follows: Forward: 5′-TTGGTTGTGGGAAGTCGT-3′ and reverse 5′-GCTTATGTAGTTAATGGGAGGT-3′ and their amplification product length was 415 bp. The GAPDH primer sequences were as follows: Forward 5′-GAGTCAACGGATTGGTCGT-3′ and reverse 5′-GACAAGCTTCCCGTTCTCAG-3′ and their amplification product length was 185 bp. The RT-qPCR parameters were as follows: 95°C pre-denaturation for 5 min, 95°C denaturation for 30 sec, 60°C annealing for 30 sec and 72°C elongation for 60 sec, for 35 cycles in total. The RT-qPCR products were detected using 1.2% agarose gel electrophoresis and the results were observed and images were captured using a UVItec gel documentation and analysis system (Mr Thaksin Technology Development Co., Ltd, Beijing, China). The gray value of the bands were analyzed using Image-Pro Plus 7.0 software (Media Cybernetics, Inc., Rockville, MD, USA) and the relative mRNA expression level of ABCE1 was expressed as ABCE1/GAPDH.

### Western blot analysis

The cells (1×10^7^) in each group were collected and washed three times in PBS. The total protein was extracted and its concentration was measured using a bicinchoninic acid assay (Advanced Magnetic, Cambridge, MA, USA). The total protein (60 *μ*g) was separated by 10% SDS polyacrylamide gel electrophoresis (PAGE; Gibco-BRL, Carlsbad, CA, USA) and electrotransformed onto a PVDF membrane using a semidry method. Membranes were blocked with 5% non-fat milk then incubated with ABCE1 polyclonal primary antibody (1:1,000) at 4°C overnight. The membrane was washed four times using Tris-buffered saline and Tween 20 (Beijing Noble Rider Technology Co., Ltd, Beijing, China) for 15 min each time. HRP-labeled secondary antibody and GAPDH (1:5,000) were added to the membrane, which was incubated at 37°C for 2 h. The membrane was washed with PBS and chemiluminescence detection was performed using an ECL kit. An X-ray film was squash slided, developed and fixed, following the exposure of X-ray film in a dark room. The gray values of the bands was analyzed using a UVI gel imaging system and Image-Pro Plus 7.0 software. The relative MRNA expression levels of ABCE1 were expressed as ABCE1/GAPDH.

### Cell cycle analysis using flow cytometry

The cells in each group were collected and their concentration was modulated to 1×10^6^/l dilution using PBS, and subsequently the cells were washed twice in cold PBS. The cell deposit was mixed with 70% ice ethanol at 4°C, washed and incubated at a concentration of 1×10^6^/m with Tris-HCL buffer containing 50 *μ*g/ml RNase (pH 7.4) for 30 min. The DNA in the cells was stained using 100 *μ*g/ml (1 *μ*g/ml^−1^) propidium iodide (PI), preserved at room temperature in the dark for 30 min. The cell cycle and apoptosis was determined by flow cytometry. The procedure was repeated three times.

### Determination of cell proliferation using the CCK-8 method

The cells in the exponential phase were inoculated in 96-well culture plates (4,000/well) and 200 *μ*l DMEM containing 10% FBS was added, with six duplication wells for each group and an empty well (without cells) as a control. CCK-8 (20 *μ*l) was added to each well. Following incubation for 4 h, the absorbance value at 490 nm was detected using an enzyme-labeled instrument (Molecular Devices, Sunnyvale, CA, USA). A growth curve was then plotted using the average absorbance value of the cells against the time points of 48, 72, 96 and 120 h.

### Wound-healing assay

The three groups of cells in the logarithmic phase were inoculated separately into 6-well plates at a density of ×10^3^ cells/well. When the cultured cells of each group had grown to 90% confluence, a cross line nick was made on the single cell layer in each group using 10 *μ*l pipette tips or a sterilized toothpick. The suspended cells were removed by washing three times with 0.01 mol/l PBS. The cells were then resuspended in 2 ml DMEM containing 10% FBS and incubated at 37°C in 5% CO_2_ for 48 h. The cell migration was visualized and images were captured under an Olympus IX71 inverted microscope (magnification, ×100; Olympus Corp., Tokyo, Japan).

### Transwell chamber invasion assay

Matrigel gel (BD Biosciences, Franklin Lakes, NJ, USA) was coated onto a polycarbonate microporous membrane (50 *μ*g/well). As a conditional cultivation liquid, 10% FBS was added to the lower chamber of the polymerized Transwell chambers (Shanghai Xia Yi Industrial Co., Ltd, Shanghai, China) in each group and 100 *μ*l of the EC109 cell suspension was added to the upper chamber in each treatment group (total cellular score of 3×10^5^/l). The chambers were incubated at 37°C in 5% CO_2_ for 24 h, fixed using 4% paraformaldehyde (Tianjin Chemical Reagent Factory, Tianjin, China) for 10 min and stained with 0.25% hematoxylin solution (Tianjin Chemical Reagent Factory) for 20 min. The cells, which were retained on the surface of the membrane in the upper chambers were removed using a cotton bud and the membrane was removed intact using surgical blades. The cells in the lower chambers were counted under a light microscope (magnification, ×200). A total of five transmembrane cells in randomly selected visual fields were counted separately on each membrane and the average calculated. There were three chambers in each group with three duplications.

### Statistical analysis

The results were analyzed using SPSS 16.0 software (SPSS, Inc., Chicago, IL, USA) and the statistical data are expressed as the mean ± standard deviation. Comparison among multiple groups was performed using single factor variance analysis and a q-test. P<0.05 was considered to indicate a statistically significant difference.

## Results

### siRNA inhibits the mRNA expression of ARCE1

**Figure 1:**
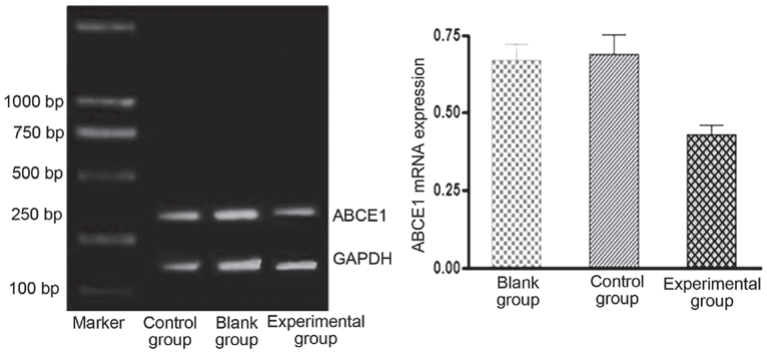
Detection of the mRNA expression of ABCE1 in the EC109 cells by reverse transcription quantitative polymerase chain reaction. Values are expressed as the mean ± standard deviation. ABCE1, ATP-binding cassette protein E1.

The results of the mRNA expression of ABCE1 are shown in Fig. 1. Compared with the control group and the blank group, the mRNA expression of ABCE1 in the EC109/ABCE1-siRNA group (experimental group) decreased significantly (P<0.05). No significant difference was observed in the mRNA expression of ABCE1 between the control group and the blank group.

### siRNA inhibits the protein expression of ABCE1

**Figure 2:**
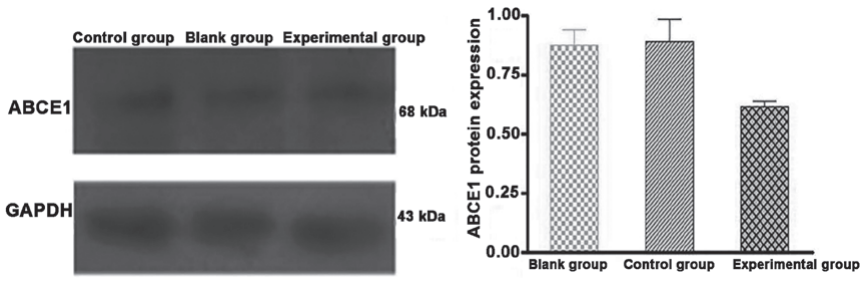
Detection of the protein expression of ABCE1 in the EC109 cells by western blot analysis. Values are expressed as the mean ± standard deviation. ABCE1, ATP-binding cassette protein E1.

The protein expression of ABCE1 are shown in Fig. 2. Compared with the control group and the blank group, the protein expression of ABCE1 in the experimental group decreased significantly (P<0.05). No significant differences were observed in the protein expression of ABCE1 between the control group and the blank group.

### Cell cycle detection

The effect of ABCE1-siRNA on the cell cycle was detected by flow cytometry. The results demonstrated that, compared with the control group and the blank group, there was a significant difference in the number of cells in the G0/G1 and S phases in the EC109/ABCE1-siRNA group (P<0.05), however, no significant difference was observed in the distribution of cells in the G0/G1 and S phases between the control group and the blank group (P>0.05), which demonstrated that ABCE1-siRNA inhibited the cell cycle at the G0/G1 phase (Table I).

### Effects on cell apoptosis

The flow cytometric analysis revealed that the apoptotic rate of the cells in the EC109/ABCE1-siRNA group were significantly higher compared with the control group and the blank group (P<0.01). No significant difference was observed in the apoptotic rate between the control group and the blank group (P>0.05; Table I).

### siRNA effects cell proliferation

**Figure 3:**
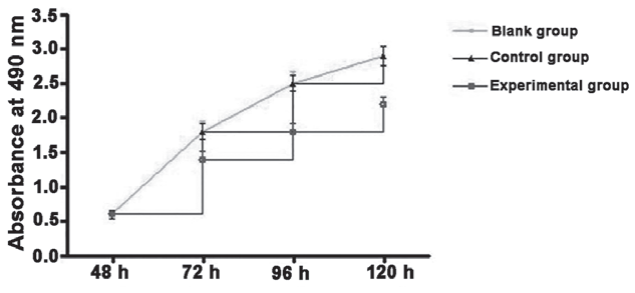
Growth curve of the three treatment groups revealing changes in absorbance with increasing duration.

The growth curve shown in Fig. 3 was produced from the results of the CCK-8 assay revealed that, compared with the control group and the blank group, the curve of the EC109/ABCE1-siRNA group was significantly decreased (P<0.05; Table II).

### Cell nick wound-healing assay

**Figure 4:**
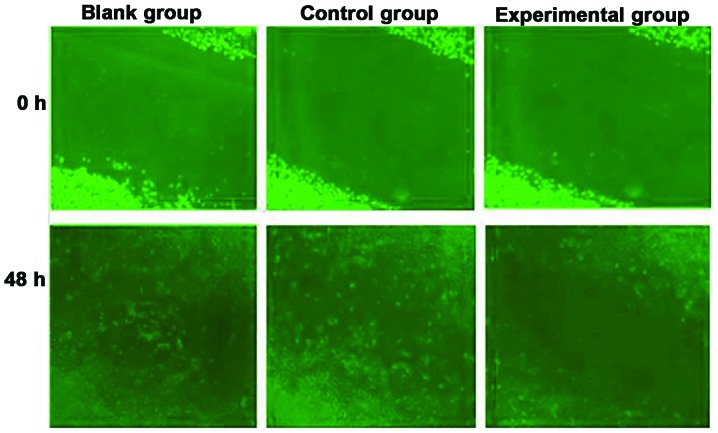
Wound-healing assay results following scratch injury. After 48 h, the cells in the experimental group exhibited relatively slow healing, however, the cells in the control group and the blank group had almost entirely covered the scratches, indicating that cell migration in the experimental group was decreased significantly.

The wound healing was observed 48 h after performing the wounding procedure. The wound healing of the cells in the EC109/ABCE1-siRNA group was slow compared with the wounds in the cell layers of the control group and blank group, which had almost healed(Fig. 4).

### Variation in the invasive capacity of EC109 cells in vitro

**Figure 5:**
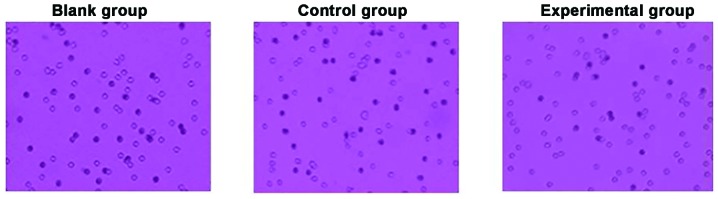
Transwell chamber invasion assay. After 48 h, the number of cells which transferred through the membrane in the experimental group was significantly lower compared with the number in the control group and the blank group (42.56±4.68; P<0.05).

As shown in Fig. 5, the number of cells that crossed the membrane in the control group and blank group were 69.45±5.84 and 70.36±6.23, respectively, which were significantly higher than the number of transmembrane cells in the EC109/ABCE1-siRNA group (42.56±4.68; P<0.01). This result demonstrated that specific interference on the gene expression of ABCE1 can effectively reduce the invasive capacity of EC109 cells

## Discussion

Electroporation is a technology, which has been developed in the last 20 years and benefits from repeatability, high efficiency, large scale sample treatment, no toxicity and the ability to control experimental parameters (7). Electroporation provides a good method of transfection in cells that are available to conventional transfection methods and is considered the superior molecular transmitting system (8). Tumor occurrence is a process with multiple stages of development, in which cells develop into a malignant tumor through a series of progressive changes (9). Inhibiting cancer genes or regulating molecular expression by electroporation may offer a novel therapy strategy.

The ABCE1 gene is located on autosomal 4q3l and a member of the APT binding cassette transporter subfamily, which is highly conserved, codes ribonuclease L protein (10) and is involved in tumor growth and development. The ABCE1 protein can inhibit the antiviral 2-5A/RNase L path in the cell, mediated by interferon, and inhibit apoptosis. There is substantial evidence that ABCE1 is closely associated with the regulation of cancer cell growth, migration and invasion and is involved in promoting cell proliferation, differentiation and protein synthesis (11). It has been demonstrated that inhibiting the expression of ABCE1 in tumor cells markedly inhibits tumor cell growth (12). Therefore, inhibiting the expression of ABCE1 in tumor cells may have an effect on tumor therapy. In order to examine the effect of ABCE1 on the migration of esophageal cancer cells in the present study, the gene expression of ABCE1 in EC109 esophageal cancer cells was inhibited by electroporation. Following gene silencing, significant decreases were observed in the rate of cell growth, the capacity of the cells to migrate and the number of cells in the S phase. In addition, the cells were inhibited at the G0/G1 phase, promoting apoptosis. In addition, nick wound-healing and Transwell chamber invasion assays revealed that, following ABCE1 gene silencing, the migration and invasive capacities of the EC109 esophageal cancer cells decreased, which was consistent with the results observed in 95-D/NCE-H466 lung cancer cells by Zhao *et al* (13).

In conclusion, the ABCE1 gene is not only closely associated with tumor cell migration and invasion, but is also important in promoting tumor cell proliferation (14). Specific interference of the gene expression of ABCE1 can inhibit the migration of EC109 esophageal cancer cells and tumor cell proliferation. Therefore, the ABCE1 siRNA sequence may be an effective target site for esophageal cancer therapy and the inhibition of ABCE1 by electroporation may offer a novel therapeutic method in the treatment of malignant tumors, including esophageal cancer.

## Figures and Tables

**Table I:** Cell cycle distribution and apoptotic rate.

Group	G0/G1 phase	S phase	G2/M phase	Apoptotic rate (%)
Blank	56.1±2.7	35.5±2.9	8.7±1.0	0.54±0.24
Control	54.2±2.5	36.0±2.6	9.2±1.1	0.64±0.28
Experimental	76.5±3.1^a^	16.2±1.4^a^	8.4±1.3^a^	15.46±3.12^a^

a P<0.05 or P<0.01 level of significance. Data are expressed as the mean ± standard deviation. n=6 in each group.

**Table II:** Absorbance at 490 nm in each treatment group at different time points.

Group	Time following transfection			
	48 h	72 h	96 h	120 h
Blank group	0.6±0.04	1.8±0.15	2.5±0.16	2.9±0.13
Control group	0.6±0.06	1.8±0.11	2.5±0.12	2.9±0.14
Experimental group	0.6±0.04^a^	1.4±0.12^a^	1.8±0.11^a^	2.2±0.10^a^

a P<0.05 or P<0.01 level of significance. Data are expressed as the mean ± standard deviation. n=6 in each group.
